# Kuroshio Extension and Gulf Stream dominate the Eddy Kinetic Energy intensification observed in the global ocean

**DOI:** 10.1038/s41598-025-06149-9

**Published:** 2025-07-01

**Authors:** Bàrbara Barceló-Llull, Pere Rosselló, Vincent Combes, Antonio Sánchez-Román, M. Isabelle Pujol, Ananda Pascual

**Affiliations:** 1 Institut Mediterrani d’Estudis Avançats, IMEDEA (CSIC-UIB), Esporles, Spain; 2 Collecte Localisation Satellites, Ramonville-Saint-Agne, France

**Keywords:** Physical oceanography, Physical oceanography

## Abstract

Ocean mesoscale variability, including meanders and eddies, is a crucial component of the global ocean circulation. The Eddy Kinetic Energy (EKE) of these features accounts for about 90% of the ocean’s total kinetic energy. This study investigates if the global ocean mesoscale variability is becoming more energetic by analyzing 30 years of satellite altimetric observations. We use two observational products: one constructed from a consistent pair of altimeters and another including all available missions. Our results reveal a significant global EKE strengthening of 1–3% per decade. The intensification is concentrated in energetic regions, particularly in the Kuroshio Extension and the Gulf Stream, which show EKE increases of ~ 50% and ~ 20%, respectively, over the last decade. These observations raise new questions about the impact of the Gulf Stream strengthening on the Atlantic meridional overturning circulation (AMOC) and challenge existing climate models, emphasizing the need for improved representation of small-scale ocean processes.

## Introduction

Earth has undergone anthropogenic global warming driven by the emissions of greenhouse gasses that have been released to the atmosphere by human activities since the beginning of the Industrial Revolution^1^. The ocean is the major Earth’s heat reservoir and has absorbed 90% of the anthropogenic excess heat^2^. Ocean circulation plays a key role in the global climate system by redistributing water masses and their properties, including heat and carbon, throughout the global ocean. At the same time, changes in the climate system have not only warmed the upper ocean but also altered the wind stress, heat, and freshwater fluxes that act as driving forces for ocean circulation^2,3^. Hence, climate change can modify the intricate system that constitutes the global ocean circulation^4–6^. The mesoscale circulation, defined as those motions with spatial scales between ~ 10–100 km, is an essential component of the global ocean circulation and is in many ways dynamically analogous to atmospheric weather^7^. It is constituted by a time-mean (or steady) flow and a time-varying flow, which we will refer here as the mesoscale variability and includes meanders and eddies. These features exist throughout the global ocean and transport and mix water masses and their properties over long distances and locally in depth, influencing larger and smaller-scale processes^7,8^. The kinetic energy associated with the mesoscale variability (called Eddy Kinetic Energy, EKE) represents about 90% of the total kinetic energy of the oceans^9,10^, making these features an essential component of the ocean circulation. Here, we aim to evaluate if the global ocean mesoscale variability is changing over the altimetric era through the analysis of global trends in EKE, which is a metric commonly used to determine the intensity of ocean currents. Providing knowledge on this topic is crucial for predicting the future state of our oceans and their role in the broader climate system.

Recently, several investigations have been exploring this subject using datasets that may have limitations for long-term analyses^11–14^. These studies employ data products with an inconsistent number of observations over time, potentially introducing erroneous trends to the evaluated variables^15,16^. The ocean currents of these products are derived from sea level measurements collected over the last three decades by a constellation of satellite altimeters with a varying number of missions that range from two to seven (Fig. S1). The inclusion of new satellites over time enhances the capacity to map mesoscale structures, despite the time-variable errors dependent on the number of satellites used. The altimetric gridded product obtained from these observations has been essential to understand the large-scale and mesoscale ocean circulation^17,18^, and a continuous effort is done to improve its resolution and accuracy, for instance with updated corrections and mapping parameters or techniques^19–23^, and with new satellites such as the recently launched Surface Water and Ocean Topography (SWOT) mission^24–26^. An additional altimetric product is constructed for climate applications^22^. This product includes observations from a consistent pair of altimeters, which is considered the minimum requirement for resolving mesoscale features^16^, and prioritizes the stability of the global mean sea level assuming the cost of reducing the spatial resolution. A constant number of altimeters ensures almost uniform errors over the altimetric era, with minor variations due to changes in the satellite constellation^13^. In consequence, this altimetric product is focused on the analysis of the long-term evolution of sea level, being appropriate for climate applications^22^.

**Fig. 1:**
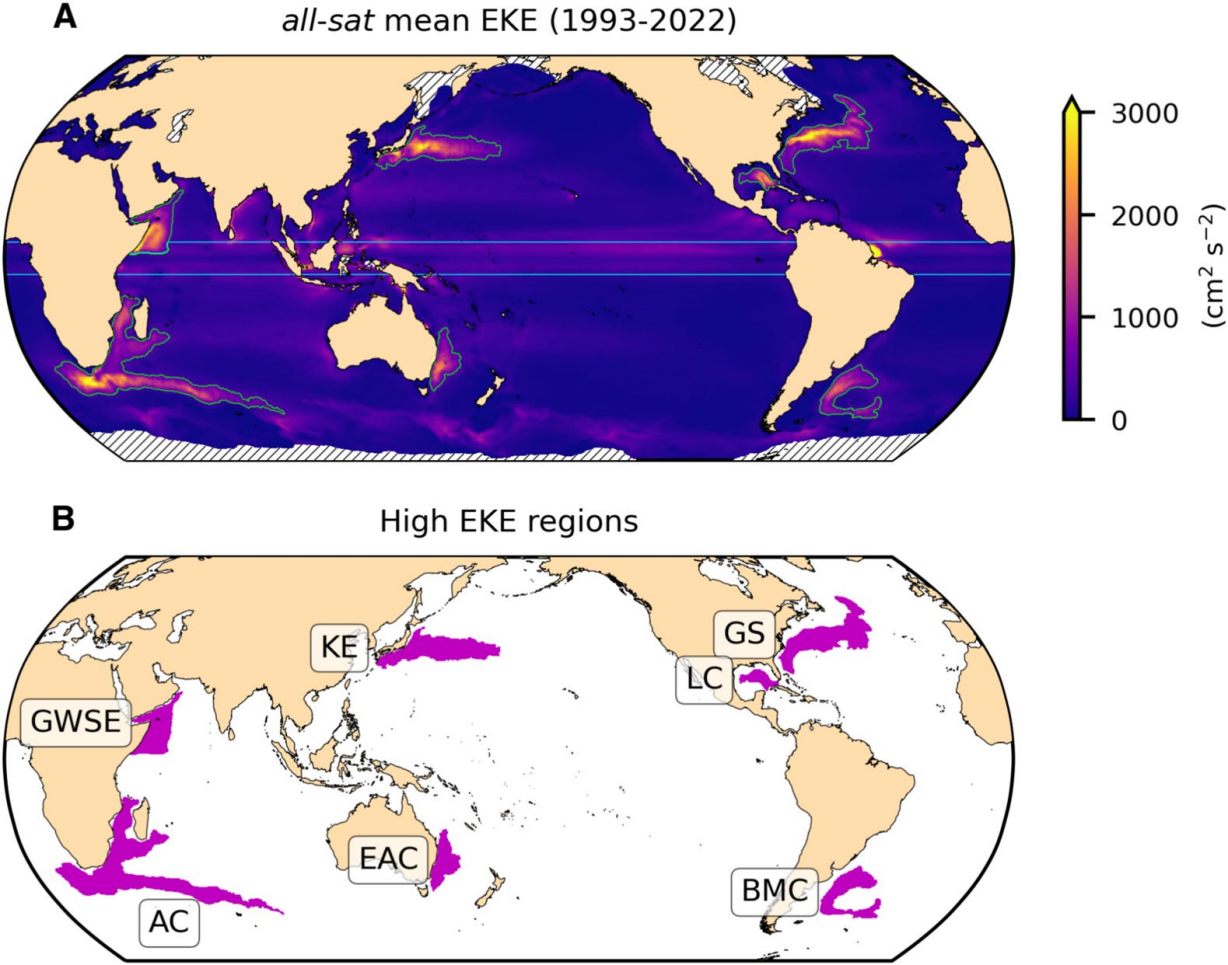
**Mean EKE and high EKE regions.** (**A**) Global map of the temporally averaged EKE field computed over the period 1993–2022 at each grid point (1/4° of spatial resolution) for the *all-sat* altimetric product. Green contours outline the high EKE regions and cyan lines delimit the tropical band. Black oblique lines indicate the areas masked due to ice coverage throughout the annual cycle. (**B**) Map showing the high EKE regions. *GWSE* Great Whirl and Socotra Eddy in East Africa, *AC* Agulhas Current, *KE* Kuroshio Extension, *EAC* East Australian Current, *GS* Gulf Stream, *LC* Loop Current, *BMC* Brazil-Malvinas Confluence region.

This study aims to assess if the global ocean mesoscale variability is becoming more energetic through the analysis of 30 years of altimetric data (1993–2022), paying special attention to regions characterized by intense mesoscale activity (Fig. 1). To address this objective, we evaluate global trends of EKE computed from altimetric observations provided by the climatic product (herein *two-sat*) and also by the product that includes all available altimeter missions (herein *all-sat*) to estimate the impact of the increasing number of satellites on the results.

## Results

### Global Eddy Kinetic Energy trends

**Fig. 2:**
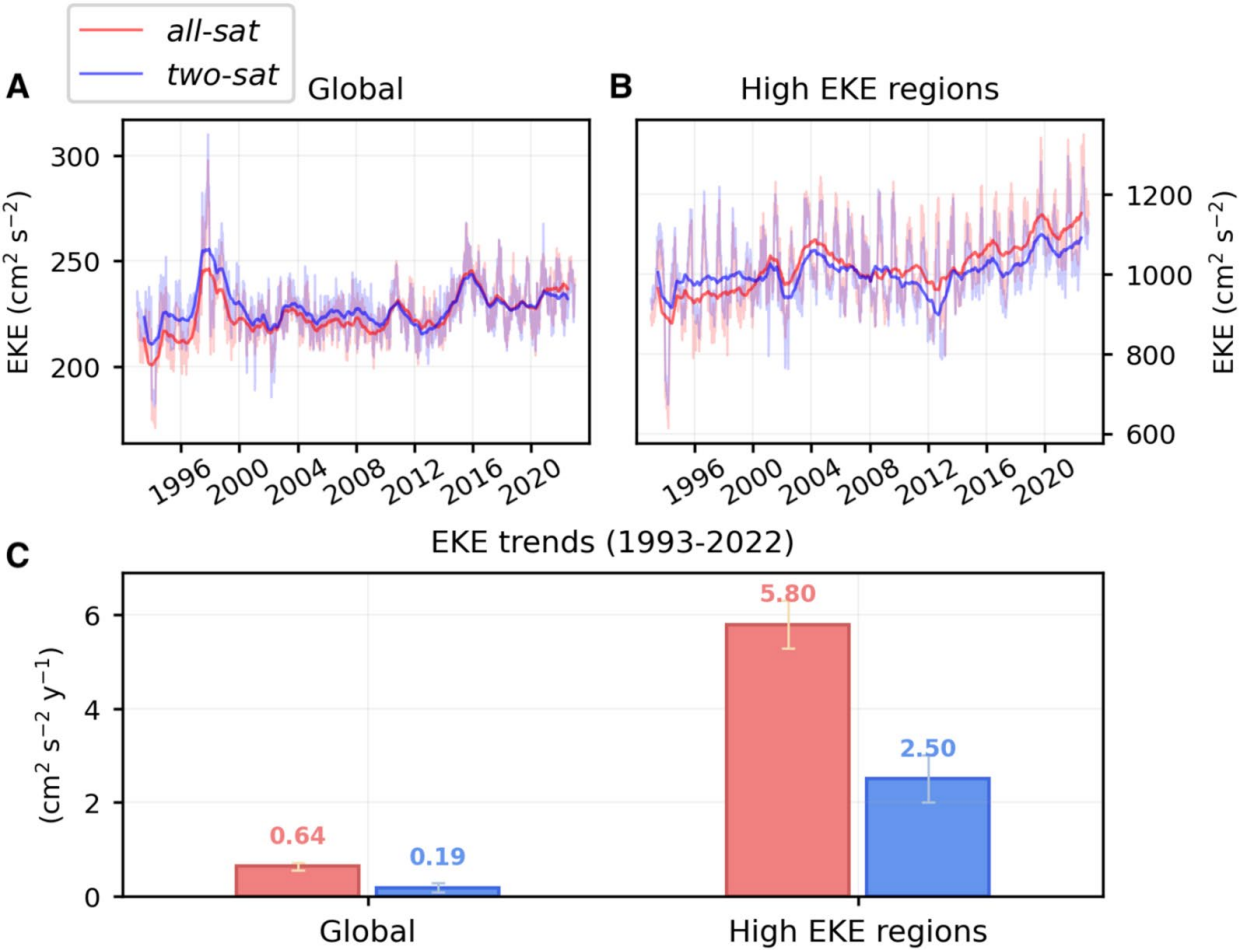
**EKE time series and trends.** Area-weighted mean EKE time series computed over (**A**) the global ocean and (**B**) the high EKE regions, for the *all-sat* (red line) and *two-sat* (blue line) altimetric products. Thinner lines represent the original data, while thicker lines show the yearly-rolling mean (i.e. 365-day-window moving average). (**C**) Trends of the original area-weighted mean EKE time series shown in (**A,B**), computed from 1993 to 2022. All trends are statistically significant (p < 0.05). Standard errors are shown with yellow (blue) error bars for *all-sat* (*two-sat)*.

**Fig. 3:**
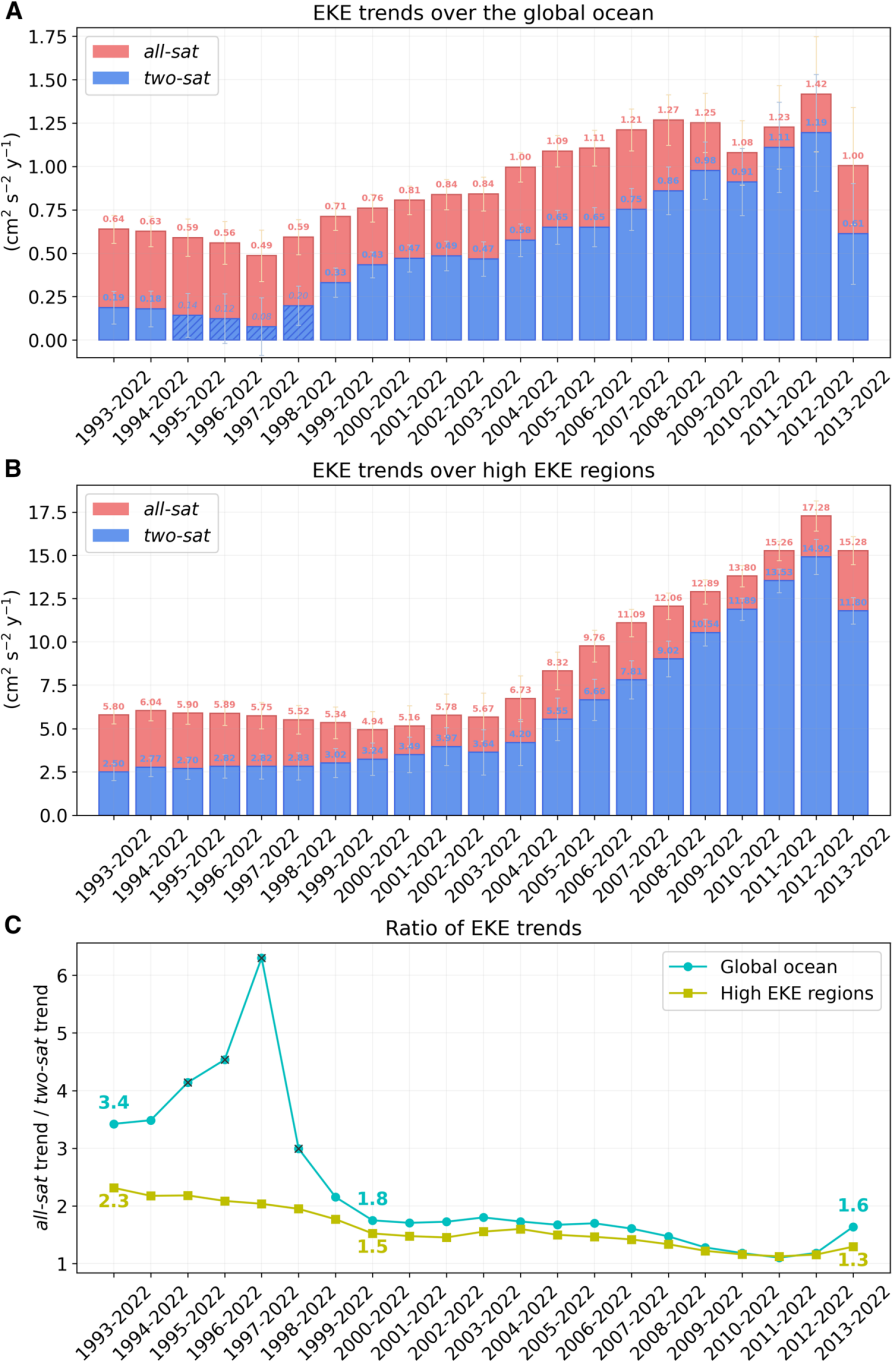
**Sensitivity test.** EKE trends computed over the (**A**) global ocean and (**B**) high EKE regions for different periods. Statistically significant trends (p < 0.05) are represented by solid-colored bars, while non-significant trends are represented as bars with oblique lines. Standard errors for *all-sat* (*two-sat*) trends are shown with yellow (blue) error bars. (**C**) Ratio of the *all-sat* EKE trend divided by the *two-sat* EKE trend.

The trends of the globally-averaged EKE time series over the altimetric era (1993–2022) are positive and statistically significant at the 95% confidence level for both altimetric products, with a large difference in magnitude (Fig. 2A,C). The trend of the *all-sat* EKE time series is 0.64 cm^2^ s^−2^ year^−1^ (or 0.066 J m^−3^ year^−1^), while the trend of the *two-sat* EKE time series is 0.19 cm^2^ s^−2^ year^−1^ (or 0.019 J m^−3^ year^−1^), i.e., 3.4 times smaller (Fig. 3C). These EKE trends represent an increase per decade of 2.8% for *all-sat* and 0.8% for *two-sat*, relative to their respective mean EKE values (Table S1). Assuming an area of the surface global ocean of 3.28 × 10^8^ km^2^, the area-integrated EKE trend is 0.22 × 10^15^ J m^−1^ decade^−1^ for the *all-sat* product and 0.06 × 10^15^ J m^−1^ decade^−1^ for the *two-sat* product. The result obtained from the *all-sat* altimetric product may suggest that the ocean mesoscale variability is experiencing a strong intensification^11,12^. However, the different result obtained from the *two-sat* product, which is based on the observations gathered by a consistent number of satellites, suggests that the larger *all-sat* EKE trend may be, at least partially, an artifact induced by the increasing number of satellites in the altimetric record^13,14^ (Fig. S1). The inclusion of additional satellites within the altimetric record can enhance the capacity to detect higher energy levels. Consequently, a progressive increase in the satellite count over time may give rise to an increase of energy attributed to this phenomenon. Another reason for this difference could be that *two-sat* may not completely capture a potential increase in mesoscale kinetic energy due to its lower resolution. Hence, both altimetric products support the conclusion that the global ocean mesoscale variability is becoming more energetic over the altimetric era, although the magnitude may be overestimated by *all-sat* and underestimated by *two-sat*.

A sensitivity test reveals that the ratio between the global *all-sat* EKE trend and the global *two-sat* EKE trend is reduced from 3.4 between 1993–2022 to 1.8 when computed for the period 2000–2022, and this value is maintained or slightly smaller for the periods evaluated afterwards (Fig. 3A,C). The year 2000 marks the division between two distinct periods in the altimetry era: an initial period characterized by a varying number of satellites ranging from 2 to ~ 3, followed by a second period where the number of satellites was consistently higher than 2 (with the exception of one month in 2008) (Fig. S1). This result indicates that the difference between *all-sat* and *two-sat* EKE trends is partly related to the varying number of satellites included to build the *all-sat* product, and this difference is higher when computing trends over the complete altimetry era and reduced, but still important, when computing trends after 2000.

A striking feature is that *two-sat*, whose limitations are not time-dependent, show a clear increase of the global EKE trend for the periods evaluated in the sensitivity test (Fig. 3A). Considering the complete altimetric era, the global *two-sat* EKE trend is 0.19 cm^2^ s^−2^ year^−1^, but this trend gets larger when shortening the time series, to a maximum of 1.19 cm^2^ s^−2^ year^−1^ over 2012–2022. Hence, both altimetric products reveal an intensification of the global ocean mesoscale variability, and *two-sat* demonstrates that this increase in EKE is much faster when evaluating the last two decades. The EKE trend computed over 2012–2022 for *two-sat* is 6.2 times larger than the trend computed over 1993–2022, while for *all-sat* this ratio is only 2.2 (Fig. 3A). The smaller ratio for *all-sat* is related to the impact that the varying number of satellites included in this product has on the computation of EKE trends. When considering the complete altimetric era, the number of satellites drastically changes from ~ 2 satellites between 1993–2000, to ~ 3–4 satellites between 2000–2016, and to higher than 4 satellites after 2016 (Fig. S1). This increase over time on the number of satellites implies an enhancement on the capacity to detect energy. A progressive increase on the satellite count could lead to an increase of the energy observed related to this phenomenon, rather than a real increase of energy in the ocean. This circumstance would result in an overestimation of the EKE trends computed from *all-sat*. However, this overestimation diminishes when analyzing shorter, more recent time periods, because this phenomenon decreases if we exclude the first years of data computed from only ~ 2 satellites.

Martínez-Moreno et al.^12^ reported a trend of the global surface area-integrated EKE of (0.09 ± 0.04) × 10^15^ J m^−1^ decade^−1^ using a previous version (vDT2018) of the *all-sat* altimetric product and following a similar methodology but computing trends over the period between 1 January 1993 to 7 March 2020 and from a smoothed 365-day running average time series. The *all-sat* EKE trend computed here over 1993–2022 from the currently available vDT2021 version is 2.4 times that value (Fig. 2). We have also calculated the *all-sat* EKE trend over the same period as Martínez-Moreno et al.^12^ and over a 365-day running average (Fig. S2), without finding large differences with respect to the trends calculated from the original data over 1993–2022 (Fig. 2). This suggests that the discrepancy between the trends computed in this study and the trends reported by Martínez-Moreno et al.^12^ is related to the version of the product used. We cannot test this sensitivity because the *all-sat* vDT2018 altimetric product is no longer available. However, the *two-sat* vDT2018 altimetric product is still available on the Copernicus Climate Change Service (C3S)^52^ and we have found high sensitivity in the results related to the version of the product analyzed (Fig. S3). Indeed, a global non-significant EKE trend of −0.002 cm^2^ s^−2^ year^−1^ was obtained for the *two-sat* vDT2018 altimetric product in comparison to the 0.114 cm^2^ s^−2^ year^−1^ non-significant trend obtained for the same period with *two-sat* vDT2021 (Fig. S3).

### Eddy Kinetic Energy trends over regions of intense mesoscale activity

The mesoscale variability intensification is clearly concentrated in regions characterized by high EKE levels (Figs. 1, 2, Fig. S4). In contrast, the Tropics and the rest of the ocean exhibit predominantly statistically non-significant trends in the averaged EKE time series (Fig. S4). The distinct peaks detected in global and tropical time series, approximately corresponding to 1998 and 2016, were previously identified as El Niño events^12^. The EKE trend computed over high EKE regions from *all-sat* is 5.80 cm^2^ s^−2^ year^−1^ (or 0.59 J m^−3^ year^−1^), while from *two-sat* is 2.50 cm^2^ s^−2^ year^−1^ (or 0.26 J m^−3^ year^−1^) (Fig. 2B,C), i.e., 2.3 times smaller (Fig. 3C). These EKE trends represent an increase per decade of 5.7% for *all-sat* and 2.5% for *two-sat*, relative to their respective mean EKE values (Table S1). Assuming a surface area of 1.65 × 10^7^ km^2^, the area-integrated EKE trend is 0.10 × 10^15^ J m^−1^ decade^−1^ for *all-sat* and 0.04 × 10^15^ J m^−1^ decade^−1^ for *two-sat*. A previous study reported an EKE increase rate of 2.5% per decade from *all-sat* vDT2018, with a different definition of high EKE regions^12^.

In high EKE regions the sensitivity test of *two-sat* shows a progressive increase of the EKE trends computed over the periods evaluated (Fig. 3B, blue bars). The sensitivity test of *all-sat* also shows a progressive increase of EKE trends (Fig. 3B, red bars), but this increase is smaller due to the time-dependent limitations of *all-sat* explained in the previous section. Because of this, we focus here the discussion on the results obtained from *two-sat*. Over the last 20 years the *two-sat* EKE trend is slightly higher than that computed over the complete 30 years of altimetry data, but afterwards the trend increases to a maximum of 14.9 cm^2^ s^−2^ year^−1^ for the period 2012–2022, which is 6.0 times larger than the trend computed over 1993–2022. This means that the ocean mesoscale variability is becoming more energetic in high EKE regions than in other regions of the world ocean (Fig. S4), and the pace of this increase of energy is faster over the last decade (Fig. 3).

**Fig. 4:**
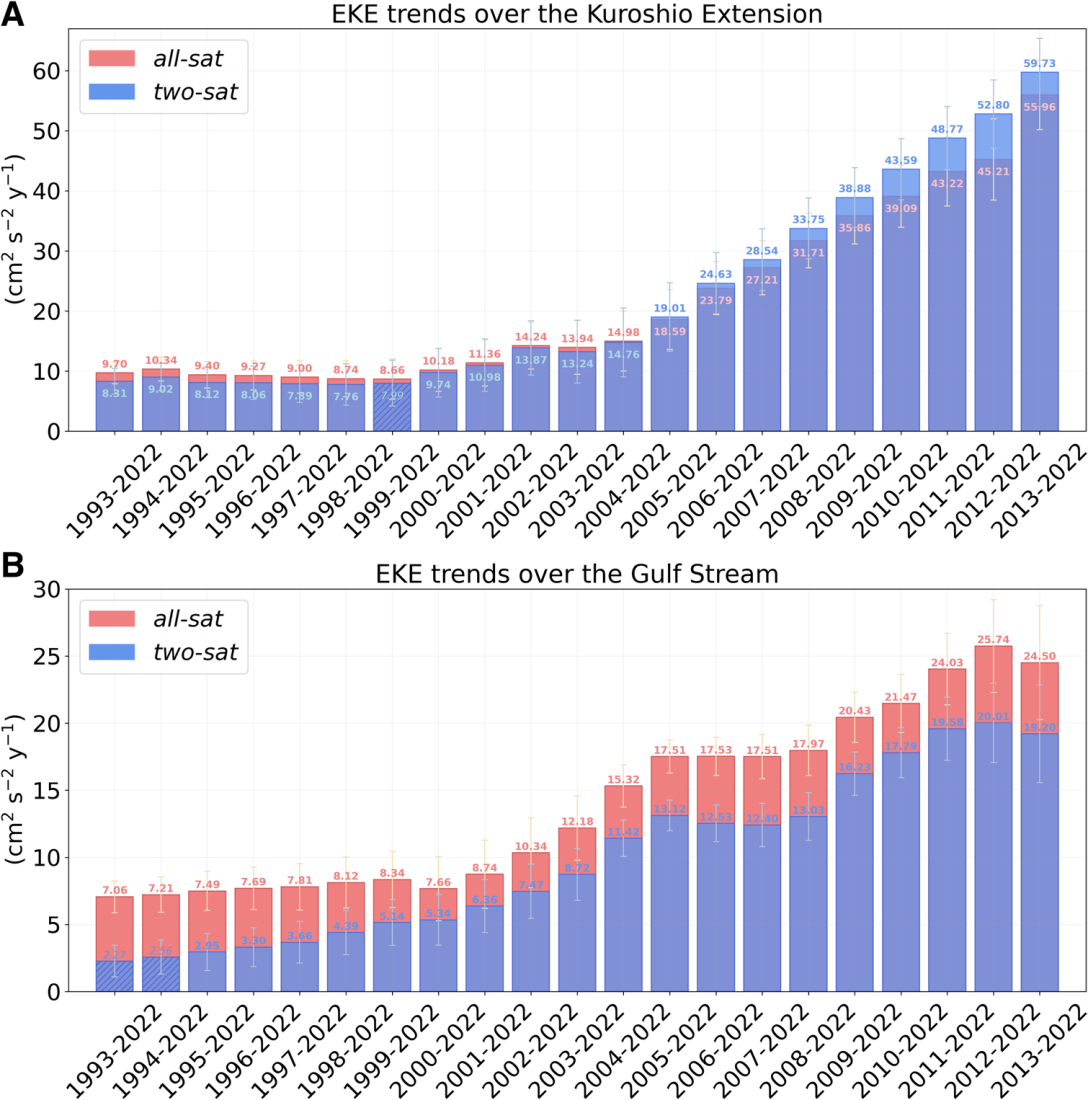
**Sensitivity test over the Kuroshio Extension and the Gulf Stream. **EKE trends computed for the (**A**) Kuroshio Extension and (**B**) Gulf Stream over different periods. Trends are computed from the original area-weighted mean EKE time series shown in Fig. S5. Statistically significant trends (p < 0.05) are represented by solid-colored bars, while non-significant trends are represented as bars with oblique lines. Standard errors for *all-sat* (*two-sat*) trends are shown with yellow (blue) error bars.

An examination of specific high EKE regions (Figs. S5, S6, Fig. 4) reveals that the domains with statistically significant positive EKE trends for both altimetric products and maintained over the periods evaluated in the sensitivity test are the Kuroshio Extension (Fig. 4A) and the Gulf Stream (Fig. 4B; with the exception of the *two-sat* 1993–2022 and 1994–2022 trends, that are not statistically significant). In the Kuroshio Extension, with both altimetric products we obtain similar EKE trends, being the ratio between the *all-sat* trend and the *two-sat* trend 1.2 for the period 1993–2022 and 0.9 between 2013 and 2022 (Fig. 4A). This suggests that in this region the impact of the varying number of satellites used to build *all-sat* is smaller than in the other regions, or even negligible, and that *two-sat*, with smaller resolution, is able to capture the magnitude of the increasing trends. Over the last 30 years, the EKE trends in the Kuroshio Extension are 9.70 cm^2^ s^−2^ year^−1^ for *all-sat* and 8.31 cm^2^ s^−2^ year^−1^ for *two-sat*, while over the last 10 years these trends are 55.96 cm^2^ s^−2^ year^−1^ and 59.73 cm^2^ s^−2^ year^−1^, respectively (Fig. 4A). This result indicates that over the last decade the EKE in the Kuroshio Extension has increased 6 to 7 times faster than over the last three decades. The EKE trends computed over the altimetric era represent an increase per decade relative to their respective mean values (Table S1) of 9.3% for *all-sat* and 8.1% for *two-sat*, while the EKE trends computed for the last decade represent an increase of 54% and 58%, respectively. In addition, the Kuroshio Extension is the high EKE region with the largest trends from both altimetric products (Figs. S5, S6, Fig. 4). A poleward migration and intensification of the Kuroshio Extension system has been previously reported from datasets that use the *all-sat* altimetric product and from climate models, revealing a relation between the EKE evolution and the Pacific Decadal Oscillation^27,28^. The analysis of EKE trends conducted here corroborates the intensification detected previously in the Kuroshio Extension.

The Gulf Stream is the second region with the largest EKE trends (Figs. S5, S6, Fig. 4). Over the last three decades, the EKE trends are 7.06 cm^2^ s^−2^ year^−1^ for *all-sat* and a statistically non-significant 2.27 cm^2^ s^−2^ year^−1^ for *two-sat*, representing a ratio of 3.1 (Fig. 4B). These trends increase to 24.50 cm^2^ s^−2^ year^−1^ for *all-sat* and 19.20 cm^2^ s^−2^ year^−1^ for *two-sat* (both trends are statistically significant) over the last decade*,* showing a reduction in the ratio to 1.3 (similar to the result obtained for the global ocean, Fig. 3C). This indicates that in the Gulf Stream the *two-sat* EKE trend has increased 8.5 times faster over the last decade than over the complete altimetric era (Fig. 4B), and that the *all-sat* EKE trend evolution may be affected by the varying number of satellites (see the discussion for the global ocean). The EKE trends computed over the last 30 years represent an increase per decade relative to their respective mean values (Table S1) of 6.5% for *all-sat* and 2.1% (statistically non-significant) for *two-sat*, while the EKE trends computed over the last decade represent an increase of 23% and 18%, respectively. A positive, consistent and increasing EKE trend in the Gulf Stream is a new result that contrasts with previous climate studies. The Gulf Stream contributes to the Atlantic meridional overturning circulation (AMOC) and is also the western boundary current of the subtropical North Atlantic gyre circulation. The AMOC plays a crucial role in regulating Earth’s climate and is constituted by the Gulf Stream, that transports warm and saline Atlantic water polewards, the cooling and densification of this water in the Nordic Seas, and the return of the cooled water at depth^29^. Previous climate studies reported a weakening of the AMOC (e.g.^30,31^). Our study raises a new question regarding how the observed strengthening of the Gulf Stream mesoscale variability influences the temporal evolution of the AMOC. In the Southern Ocean, experiments with high-resolution ocean models reveal that mesoscale eddies may mitigate the effects of a warming climate by maintaining the strength of the Antarctic Circumpolar Current^32^ and delaying the decline of Antarctic sea ice^33^. These processes, together with the increase of EKE detected in the Gulf Stream, occur at scales smaller than those resolved in climate models. Additionally, submesoscale dynamics, not resolved by nadir altimeters, may transfer kinetic energy to mesoscale eddies, contributing to the reported EKE intensification (e.g.^34^). To study the impact of small-scale ocean processes in the large-scale climate system we need to better represent them in climate models and projections, or use alternative approaches^35^. In addition, the relationship between the Gulf Stream, the AMOC and the subtropical North Atlantic gyre is complex and makes necessary a sustained long-term observation of the ocean and the development of novel techniques to analyze all available data comprehensively^36^. The study conducted here highlights the importance of analyzing the relation between the strengthening of the mesoscale variability in the Gulf Stream and the temporal evolution of the subtropical North Atlantic gyre and AMOC.

Chi et al.^37^ evaluated 26 years of along-track altimetric data (1993–2018) to determine if the expected deceleration and poleward shift of the Gulf Stream by climate predictions were observable. They calculated linear trends in several metrics (latitude, transport, width and maximum downstream velocity) in stream-following coordinates and concluded that the trends were not significant. They also mentioned that the only locations with trend confidence showed that the Gulf Stream had accelerated and narrowed. Another study based on satellite observations revealed a correlation between mesoscale variability and the Gulf Stream meridional position^38^. A more energetic mesoscale field was associated with a northward shift of the Gulf Stream position and with a positive North Atlantic Oscillation (NAO).

A recent study by Sánchez-Román et al.^39^ provides additional insight into Gulf Stream dynamics. They examined 30 years of altimetric data (1993–2022) and an ocean reanalysis product to investigate the evolution of the Gulf Stream destabilization point—the location where the stable jet transitions into an unstable, meandering path. They observed a significant westward and southward shift of the destabilization point until 2012, followed by a reversal to an eastward and northward migration through 2022. This northeastward migration was associated with an increase in EKE and an acceleration of surface geostrophic velocities. Their analysis revealed a strong correlation between the displacement of the destabilization point and NAO variability, indicating that the Gulf Stream path may respond to NAO-driven changes over time. The findings by Sánchez-Román et al.^39^ align with our results in the Gulf Stream. Together, these studies highlight the dynamic nature of the Gulf Stream system and its sensitivity to both internal variability and external forcing. This reinforces the need for continued monitoring to understand how Gulf Stream dynamics may evolve under future climate scenarios.

The positive and increasing EKE trends obtained in the Kuroshio Extension and the Gulf Stream are in opposition to the non-significant trends reported by Martínez-Moreno et al.^12^ from the *all-sat* vDT2018 altimetric product (their Fig. 6 in Extended Data). A possible reason for this difference may be the distinct methodologies followed to define the high EKE regions. They define them with the 99th percentile of the mean kinetic energy (computed from the time-mean velocity field), while we use the 90th spatial percentile on the mean EKE field. We have compared the boundaries obtained from each methodology and found negligible differences (Fig. S7). Hence, the discrepancy between results comes from the older version of the altimetric product that they used and the shorter time series that was available at that moment. As the time series expands and the altimetric products are improved in the future, it will be necessary to reevaluate the analysis of the EKE trends conducted here to determine if our results remain consistent over time.

Beech et al.^40^ analyzed the long-term evolution of EKE using a climate model with variable-resolution aimed at increasing grid precision in high EKE regions. That model has the knowledged limitations of (i) underrepresenting the EKE with respect to altimetric observations (especially in high latitudes) and (ii) representing a North Atlantic EKE distribution more zonal than observed by satellite altimetry (their Fig. 2). In that study EKE is projected to shift poleward in several high EKE regions, to increase in the Kuroshio Current and to decrease in the Gulf Stream. However, their EKE representation in the Kuroshio Current is more similar to altimetric observations than the EKE representation in the Gulf Stream, which is smaller in magnitude, particularly in the northern part, and differs in position. They show that the Gulf Stream is projected to decrease in eddy activity over the twenty-first century, in opposition to the result obtained here from 30 years of satellite altimetry. This discrepancy is likely attributable to the limitations of the climate model over the North Atlantic.

## Conclusions

We have investigated the Eddy Kinetic Energy (EKE) temporal evolution to evaluate if the surface global ocean is becoming more energetic through the analysis of 30 years of satellite altimetry observations (1993–2022). Ocean mesoscale variability is a key component of the global ocean circulation and includes fronts, meanders and eddies on spatial scales between ~ 10–100 km. The EKE associated with these features accounts for about 90% of the total kinetic energy of the oceans^9,10^. We have computed EKE trends from two altimetric products: *all-sat* includes all available altimetry data and is constructed to study mesoscale dynamics, while *two-sat* considers a consistent number of satellites and is built for climate applications. The globally-averaged EKE time series over the altimetric era (1993–2022) show statistically significant positive trends, with the *all-sat* product indicating a larger increase compared to the *two-sat* product. Our results suggest that the increasing number of satellites in the altimetric record may partly contribute to the observed differences. Despite this, both altimetric products support the conclusion that the global ocean mesoscale variability is strengthening, and this intensification is concentrated in regions characterized by high EKE levels.

Robust statistically significant positive EKE trends are observed in the Kuroshio Extension and the Gulf Stream. The Kuroshio Extension has the largest EKE trends from both altimetric products. Over the last three decades, the EKE trends in the Kuroshio Extension are 9.70 cm^2^ s^−2^ year^−1^ for *all-sat* and 8.31 cm^2^ s^−2^ year^−1^ for *two-sat*, indicating an intensification of EKE of ~ 8–9% per decade with respect to mean values. The trends in this region are similar for both datasets, suggesting that the impact of the varying number of satellites used to build *all-sat* is smaller than in the other regions. Over the last decade, the EKE in the Kuroshio Extension has increased 6 to 7 times faster than over the last three decades, representing an increase of ~ 50% with respect to mean values. These findings support previous studies that detected an intensification of the Kuroshio Extension, potentially linked to the Pacific Decadal Oscillation.

The Gulf Stream is the second region with the largest EKE trends. Over the altimetric era, the EKE trends in the Gulf Stream are 7.06 cm^2^ s^−2^ year^−1^ for *all-sat* and a statistically non-significant 2.27 cm^2^ s^−2^ year^−1^ for *two-sat* (note that for *two-sat* non-significant trends are obtained only for the periods 1993–2022 and 1994–2022, being significant for all the other periods evaluated in the sensitivity test), representing an increase per decade relative to their respective mean values of 6.5% for *all-sat* and 2.1% for *two-sat*. Over the last decade, this region has increased 8.5 times faster than over the complete altimetric era, representing a statistically significant increase of ~ 20% with respect to mean values. A positive, consistent and increasing EKE trend in the Gulf Stream opens new questions about its relationship with the Atlantic meridional overturning circulation (AMOC) and the subtropical North Atlantic gyre. Sustained long-term observation of the ocean and the development of novel techniques to analyze all available data exhaustively are necessary to study the complex relationship between the Gulf Stream, the AMOC and the subtropical North Atlantic gyre.

The observed strengthening of mesoscale variability in the Gulf Stream challenges existing climate model projections. Our findings emphasize the need for improved representation of small-scale ocean processes in climate models to better understand their influence on the large-scale climate system. A comprehensive analysis of the dynamics driving changes in mesoscale variability is necessary to discern anthropogenic change from natural variability. Our results are also relevant for studies that use models with assimilation of observations or that rely on observations for their validation, as considering an expanding set of observations over time may lead to overestimated trends^14^.

As the altimetric record increases and future advancements enhance altimetric products, it will be necessary to reassess the analysis of EKE trends conducted here to verify the consistency of our findings over time. The Surface Water and Ocean Topography (SWOT) mission^25,26^ provides high-resolution altimetric observations that offer unprecedented opportunities to investigate the contribution of submesoscale processes to the observed intensification of EKE^41,42^. Validation of SWOT data through multi-platform in situ observations is essential to ensure the accuracy of EKE estimates at these small scales^43–45^. The long-term maintenance of the altimetric satellite constellation will be crucial to evaluate EKE trends and better understand the evolving energetics of the global ocean.

## Data and methods

### Altimetry data products

In this study, we use the latest version of the global multi-satellite Delayed Time (DT) Data Unification and Altimeter Combination System (DUACS)^13,46^, named vDT2021 and freely available through the European Copernicus Program (https://marine.copernicus.eu/). The vDT2021 product supersedes the previous vDT2018 version when comparing with independent in situ observations^22^. The DUACS system generates two distinct types of altimetric Level-4 (L4) gridded products for the global ocean: the *all-sat* and the *two-sat* products. The *all-sat* product^13^, disseminated via the Copernicus Marine Service (CMEMS project (Product ID: SEALEVEL_GLO_PHY_L4_MY_008_047, 10.48670/moi-00148), incorporates all available altimeters at a given time, ranging from 2 to 7 over the altimetric period (Fig. S1). It emphasizes the mesoscale mapping capacity of the altimeter data and the stability of the overall dataset, despite the time-variable errors dependent on the number of satellites used^13^. The *two-sat* product^46^, distributed via the Copernicus Climate Change Service (C3S) project and also by CMEMS (Product ID: SEALEVEL_GLO_PHY_CLIMATE_L4_MY_008_057; 10.48670/moi-00145), is derived from a consistent pair of altimeters, which is considered the minimum requirement for retrieving mesoscale signals in delayed time conditions^16^. The *two-sat* product is mainly based on the long-term TOPEX/POSEIDON/Jason orbit and completed by a second mission on the ERS/Envisat/AltiKa or the more recent Sentinel-3 orbit^18^. This product prioritizes the stability of the global mean sea level, assuming the cost of reducing the spatial coverage of the ocean. The steady number of altimeters ensures nearly consistent errors throughout the entire time period, barring minor variations due to changes in the satellite constellation^13^. The *two-sat* product is aimed at monitoring the long-term evolution of sea level, therefore it is appropriate for climate studies of sea level (large-scale signals)^22^.

The validation of altimetry products is a fundamental step in the DUACS data processing to assess and characterize the errors associated with the altimetry measurements^47^. The quality of both *all-sat* and *two-sat* altimetric products is mainly assessed through the analysis of the sea level anomaly (SLA) field at different steps of the processing and through the evaluation of the SLA consistency along the tracks of different altimeters and between gridded and along-track products, in addition to comparisons with external in situ measurements^13^.

Both the *all-sat* and *two-sat* products provide geostrophic velocity anomalies derived from the gridded SLA field, which is calculated with respect to a temporal mean of sea surface height over the same period (1993–2012^13^). The geostrophic velocity anomalies provided by the altimetric products represent ocean currents at the surface and are computed through the application of the geostrophic approximation by using a 9-point stencil width methodology^48^ for latitudes outside the ± 5° N band. In the equatorial band, they are computed through the Lagerloef methodology^49^ with the β plane approximation. Both the *all-sat* and *two-sat* data products cover the period ranging from 1 January 1993 to 7 June 2023 (last accessed in March 2024) and have a spatio-temporal resolution of 1/4° and 1 day. To study the temporal evolution of the EKE we analyze data from complete years, i.e., from 1993 to 2022.

### Eddy Kinetic Energy computation

The calculation of the Eddy Kinetic Energy (EKE) is performed with the following expression:

$$\text{EKE }= \frac{1}{2}\uprho ({\text{u}}_{\text{a}}^{2}+{\text{v}}_{\text{a}}^{2}),$$

where ρ = 1025 kg m^−3^ is the constant approximated sea water density, and ${\text{u}}_{\text{a}}$ and ${\text{v}}_{\text{a}}$ are the zonal and meridional geostrophic velocity anomalies, respectively, provided by the altimetric products. The EKE SI units are J m^−3^. However, we will be working instead with the EKE normalized by the density, whose units are cm^2^ s^−2^, as done by the altimetric community. The relation between the two conventions is a constant factor:

$$\text{EKE }[\text{J }{\text{m}}^{-3}] \sim 0.1025 \cdot \text{ normalized EKE }[{\text{cm}}^{2}{\text{s}}^{-2}].$$

Hereinafter, the normalized EKE will be called EKE. The EKE is computed from the geostrophic velocity anomaly fields, and therefore it represents the kinetic energy associated with deviations from the mean oceanic flow.

To compute the mean EKE and the EKE trends, an ice mask is implemented to systematically exclude regions covered by ice throughout the annual cycle. Thus, the analysis is confined to latitudes between 65°N and 65°S, where the ocean remains mostly ice-free throughout the year, ensuring consistent, uninterrupted satellite altimetry measurements.

To calculate spatial averages of EKE, we compute the area-weighted arithmetic mean with the following equation:

$$\overline{\text{EKE} } =\frac{\sum_{\text{i},\text{j}}\left[{\text{ area}}_{\text{i},\text{j}} \cdot {\text{EKE}}_{\text{i},\text{j}}\right]}{\sum_{\text{i},\text{j}}{\text{area}}_{\text{i},\text{j}}},$$

where ${\text{area}}_{\text{i},\text{j}}$ is the area of each grid cell within the selected region, i represents indices along the longitude axis, and j represents indices along the latitude axis.

### Definition of high EKE regions

In this study, we delineate areas characterized by high EKE, hereinafter referred to as high EKE regions (see Fig. 1). They are identified as those regions exceeding the 90th spatial percentile on the mean EKE field computed over the period 1993–2022 from the *all-sat* data product. To avoid the potential inclusion of small patches with high EKE, we adopt a filtering process consisting of selecting only large and well-defined regions (roughly above 4 × 10^5^ km^2^ in area), resulting in high EKE regions only covering 5% of the global ocean. These regions coincide with the Gulf Stream, the Kuroshio Extension, the Agulhas Current, the Brazil-Malvinas Confluence region, the Loop Current, the Great Whirl and Socotra Eddy in East Africa, and the East Australian Current (Fig. 1).

### Computation of EKE trends

EKE trends have been computed using the Theil–Sen estimator, while the statistical significance has been calculated with the modified Mann–Kendall test, which accounts for autocorrelations within the time series^50^. The standard errors of the EKE trends have been calculated as the residual standard error divided by the square root of the sum of squared differences in the independent variable^51^, considering the effective sample size of the time series from the modified Mann–Kendall test^12,50^.

## Supplementary Information

Supplementary Information.

## Data Availability

The altimetric data products used in this study are publicly available via the following links (last accessed in March 2024). The vDT2021 *all-sat* product is available at the Copernicus Marine Service (CMEMS) website via 10.48670/moi-00148 (Product ID: SEALEVEL_GLO_PHY_L4_MY_008_047). The vDT2021 *two-sat* product is available at the CMEMS website via 10.48670/moi-00145 (Product ID: SEALEVEL_GLO_PHY_CLIMATE_L4_MY_008_057). The vDT2018 and vDT2021 *two-sat* products are available at the Copernicus Climate Change Service (C3S) website via 10.24381/cds.4c328c78.

## Code availability

The Python codes and Jupyter Notebooks to reproduce or extend this study are publicly available on Zenodo via https://doi.org/10.5281/zenodo.15655616.
