# Supplementary material for: Kuroshio Extension and Gulf Stream dominate the Eddy Kinetic Energy intensification observed in the global ocean
Source: Sci Rep. 2025 Jul 1;15:21754. doi: 10.1038/s41598-025-06149-9 (PMC12214639; doi:10.1038/s41598-025-06149-9)
Supplement: Supplementary file 1 — Supplementary Information. [file 41598_2025_6149_MOESM1_ESM.pdf]

Supporting Information for

Kuroshio Extension and Gulf Stream dominate the Eddy Kinetic Energy intensification observed in the Global Ocean

Bàrbara Barceló-Llull[1]*, Pere Rosselló[1], Vincent Combes[1], Antonio Sánchez-Román[1], M. Isabelle Pujol[2] and Ananda Pascual[1]

[1]Institut Mediterrani d'Estudis Avançats, IMEDEA (CSIC-UIB); Esporles, Spain.

[2]Collecte Localisation Satellites; Ramonville-Saint-Agne, France.

*Corresponding author: Bàrbara Barceló-Llull (bbarcelo@imedea.uib-csic.es)

Contents of this file

Figures S1 to S7
Table S1

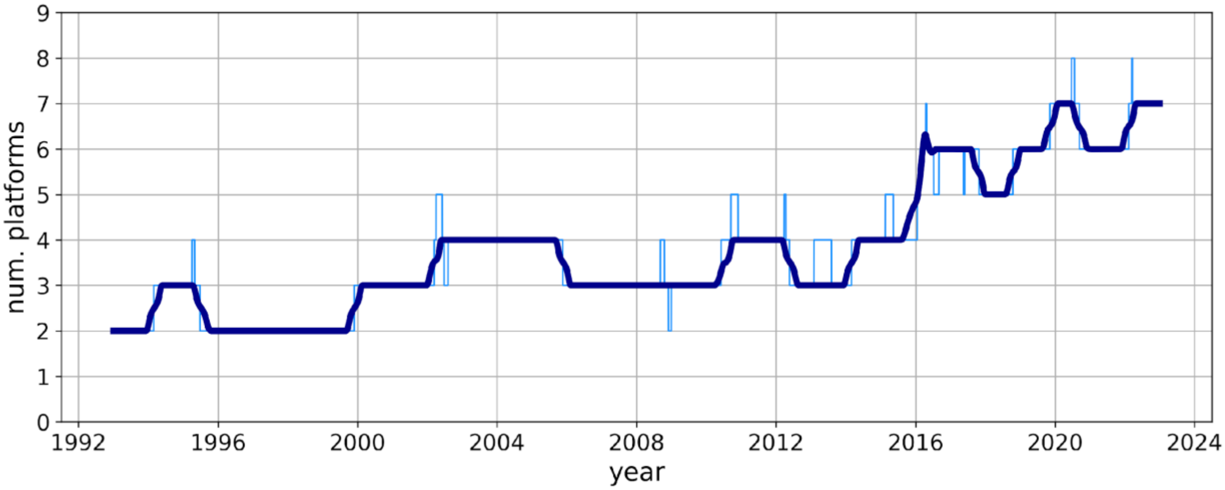

Figure S1. Number of altimetric missions. Original (pale-blue) and yearly low-pass filtered time series (blue) of the number of satellites operating at a given time used to construct the *all-sat* altimetric product.

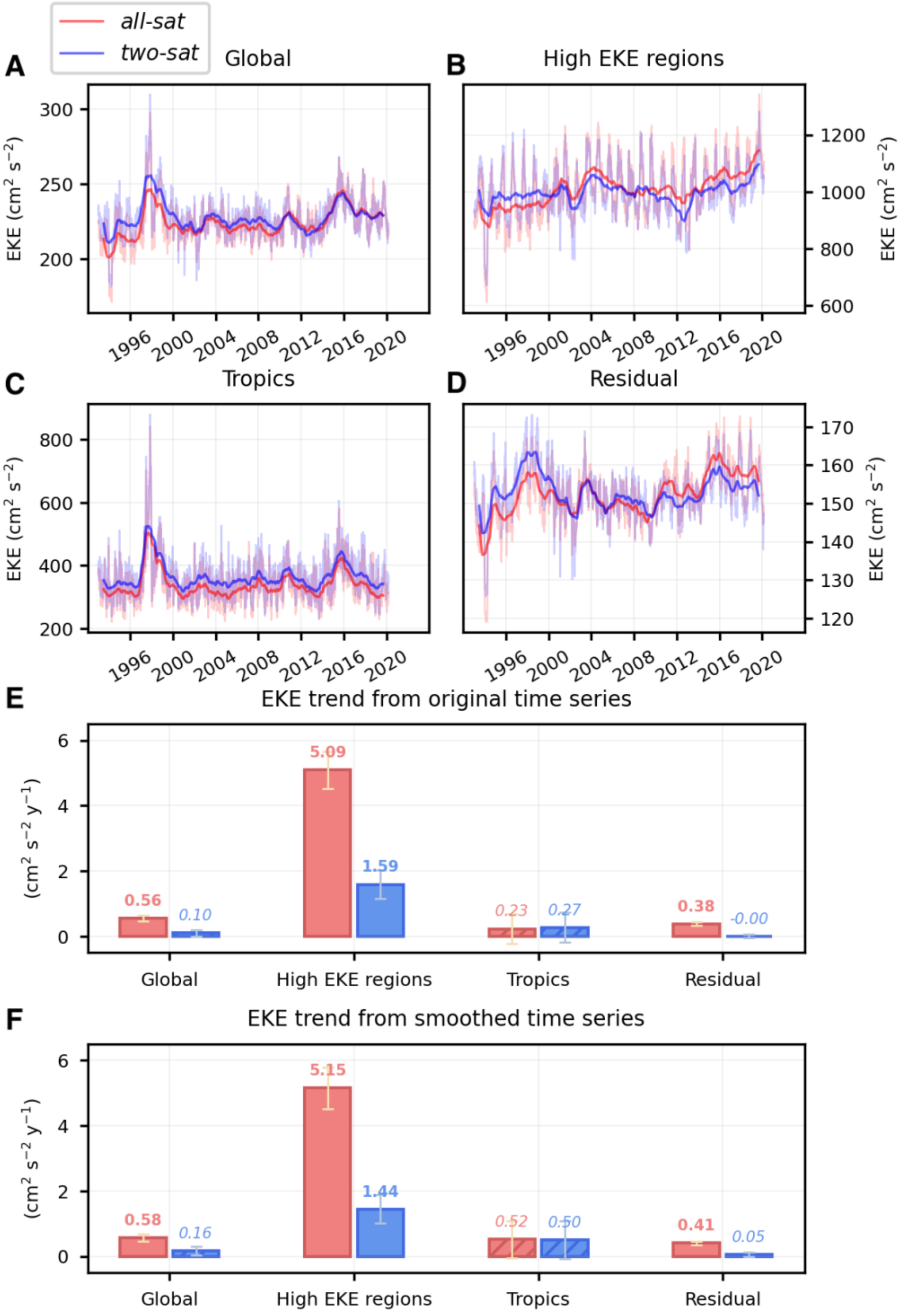

Figure S2. EKE time series and trends for the period between 1 January 1993 and 7 March 2020 (Martínez-Moreno et al., 2021). Area-weighted mean EKE time series computed over (A) the global ocean, (B) the high EKE regions, (C) the tropical band, and (D) the global ocean excluding the high EKE regions and the tropical band (called

residual), for the *all-sat* vDT2021 (red line) and *two-sat* vDT2021 (blue line) altimetric products. Thinner lines represent the original data, while thicker lines show the smoothed 365-day running average. (E) Trends of the original area-weighted mean EKE time series shown in (A-D). (F) Trends of the smoothed area-weighted mean EKE time series shown in (A-D). In (E) and (F) significant trends ($p < 0.05$) are represented by solid-colored bars, while non-significant trends are represented as bars with oblique lines. Standard errors for *all-sat* (*two-sat*) trends are shown with yellow (blue) error bars.

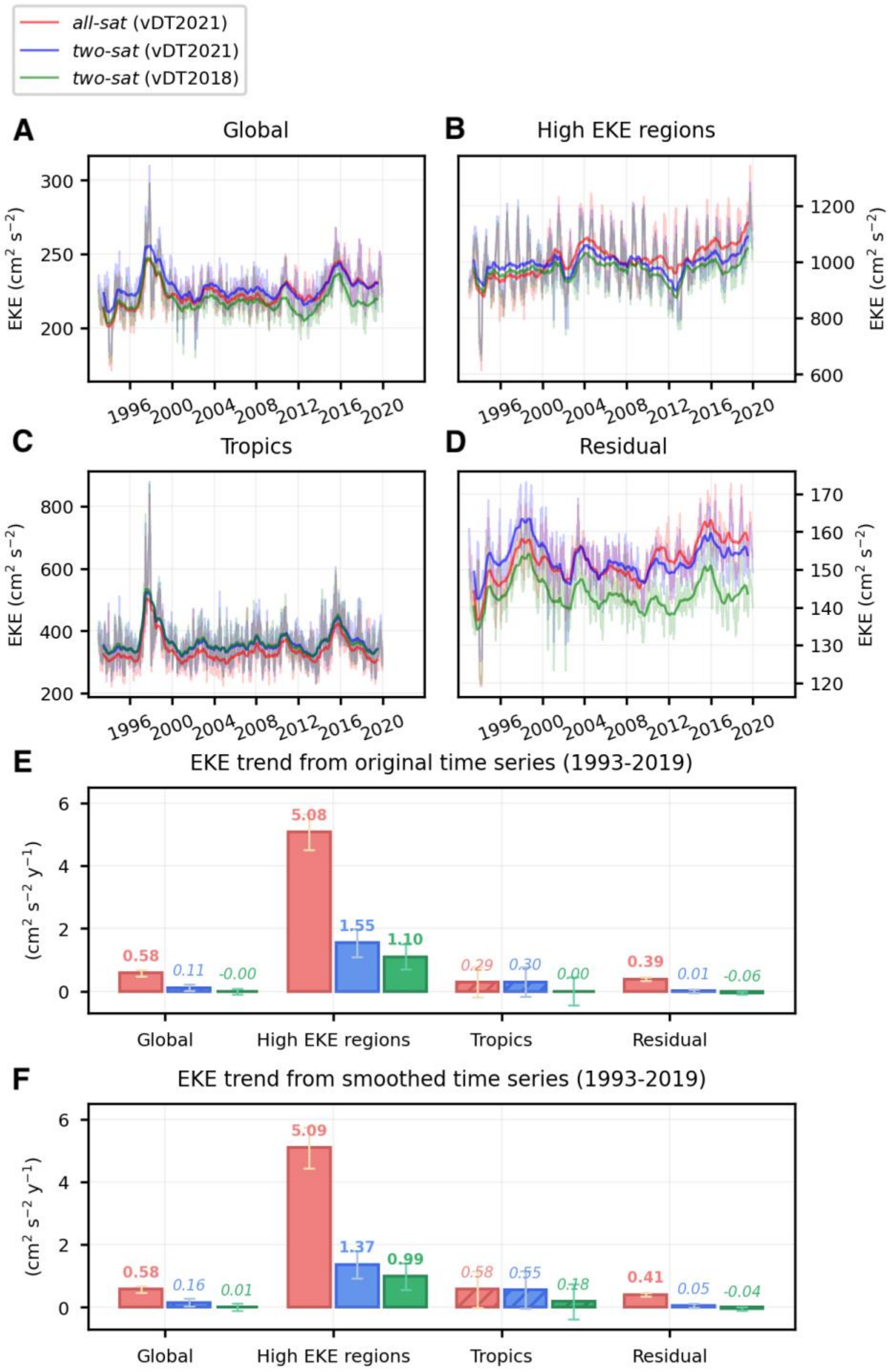

Figure S3. EKE time series and trends for three different altimetric products. EKE time series and their trends for the period between 1 January 1993 and 31 December 2019 (maximum available complete year for vDT2018) for three different altimetric products: *all-sat* (vDT2021), *two-sat* (vDT2021), *two-sat* (vDT2018). Area-weighted mean EKE time series computed over (A) the global ocean, (B) the high EKE regions, (C) the

tropical band, and (D) the global ocean excluding the high EKE regions and the tropical band (called residual), for the *all-sat* vDT2021 (red line), *two-sat* vDT2021 (blue line), *two-sat* vDT2018 (green line) altimetric products. Thinner lines represent the original data, while thicker lines show the smoothed 365-day running average. (E) Trends of the original area-weighted mean EKE time series shown in (A-D). (F) Trends of the smoothed area-weighted mean EKE time series shown in (A-D). In (E) and (F) significant trends ($p < 0.05$) are represented by solid-colored bars, while non-significant trends are represented as bars with oblique lines. Standard errors are shown with error bars.

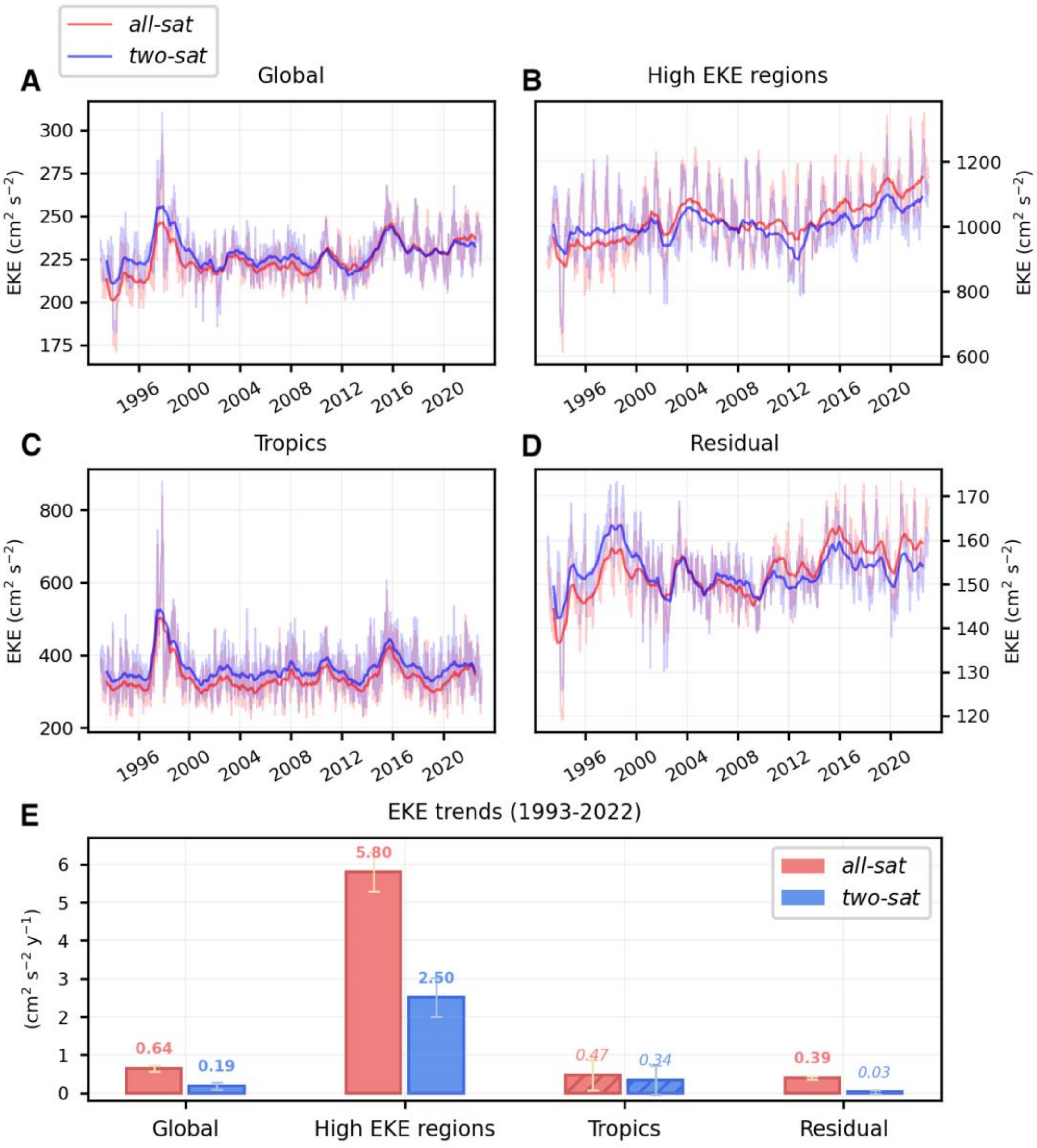

Figure S4. EKE time series and trends. Area-weighted mean EKE time series computed over (A) the global ocean, (B) the high EKE regions, (C) the tropical band, and (D) the global ocean excluding the high EKE regions and the tropical band (called residual), for the *all-sat* (red line) and *two-sat* (blue line) altimetric products. Thinner lines represent the original data, while thicker lines show the yearly-rolling mean (i.e. 365-day-window moving average). (E) Trends of the original area-weighted mean EKE time series shown in (A-D). Significant trends ($p < 0.05$) are represented by solid-colored bars, while non-significant trends are represented as bars with oblique lines. Standard errors for *all-sat* (*two-sat*) trends are shown with yellow (blue) error bars.

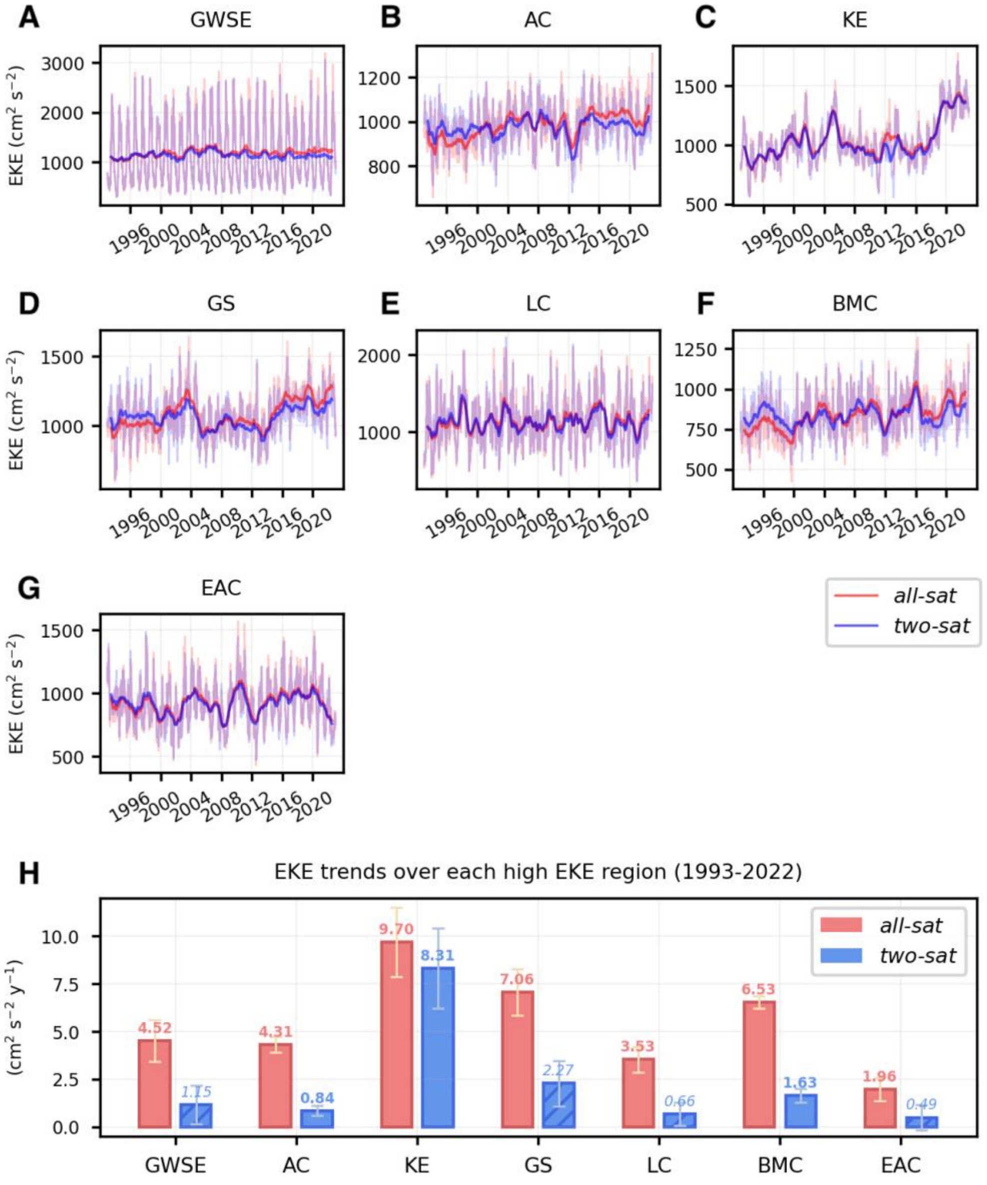

Figure S5. EKE time series and trends over each high EKE region. Comparison of the area-weighted mean EKE time series computed over each high EKE region from 1993 to 2022: (A) Great Whirl and Socotra Eddy in East Africa (GWSE), (B) Agulhas Current (AC), (C) Kuroshio Extension (KE), (D) Gulf Stream (GS), (E) Loop Current (LC), (F) Brazil Malvinas Confluence region (BMC) and (G) East Australian Current (EAC). Thinner lines represent the original data, while thicker lines show the smoothed 365-day running average. (H) Trends of the original area-weighted mean EKE time series shown in (A-G). Significant trends ($p < 0.05$) are represented by solid-colored bars, while non-significant trends are represented as bars with oblique lines. Standard errors for *all-sat* (*two-sat*) trends are shown with yellow (blue) error bars.

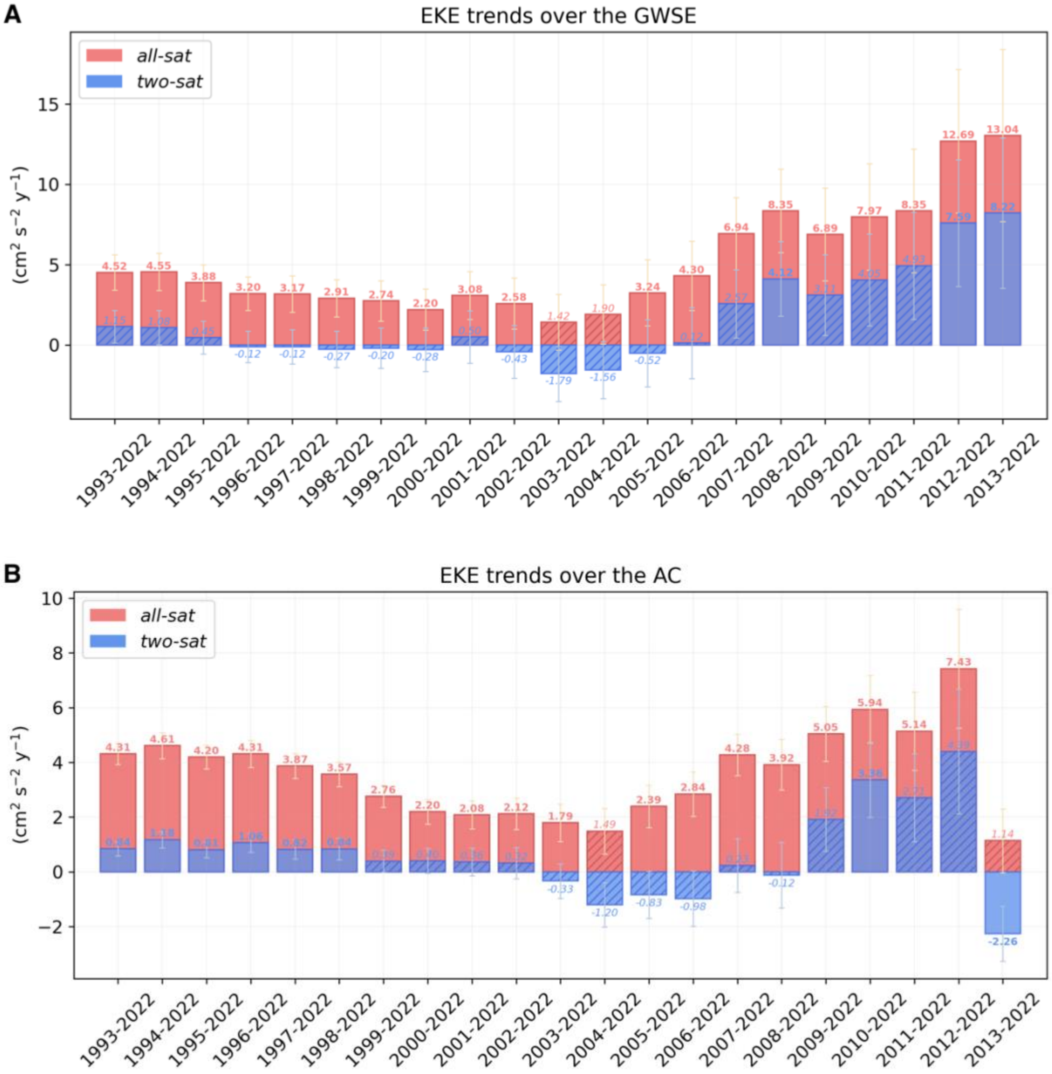

Figure S6. (This figure continues on the next page) Sensitivity test over each high EKE region. Sensitivity test of the EKE trends computed over each high EKE region for different periods (Kuroshio Extension and Gulf Stream are shown in Fig. 4). Trends are computed from the original area-weighted mean EKE time series shown in Fig. S5. Significant trends ($p < 0.05$) are represented by solid-colored bars, while non-significant trends are represented as bars with oblique lines. Standard errors for *all-sat* (*two-sat*) trends are shown with yellow (blue) error bars. (A) Great Whirl and Socotra Eddy in East Africa (GWSE), (B) Agulhas Current (AC), (C) Loop Current (LC), (D) Brazil Malvinas Confluence region (BMC), and (E) East Australian Current (EAC).

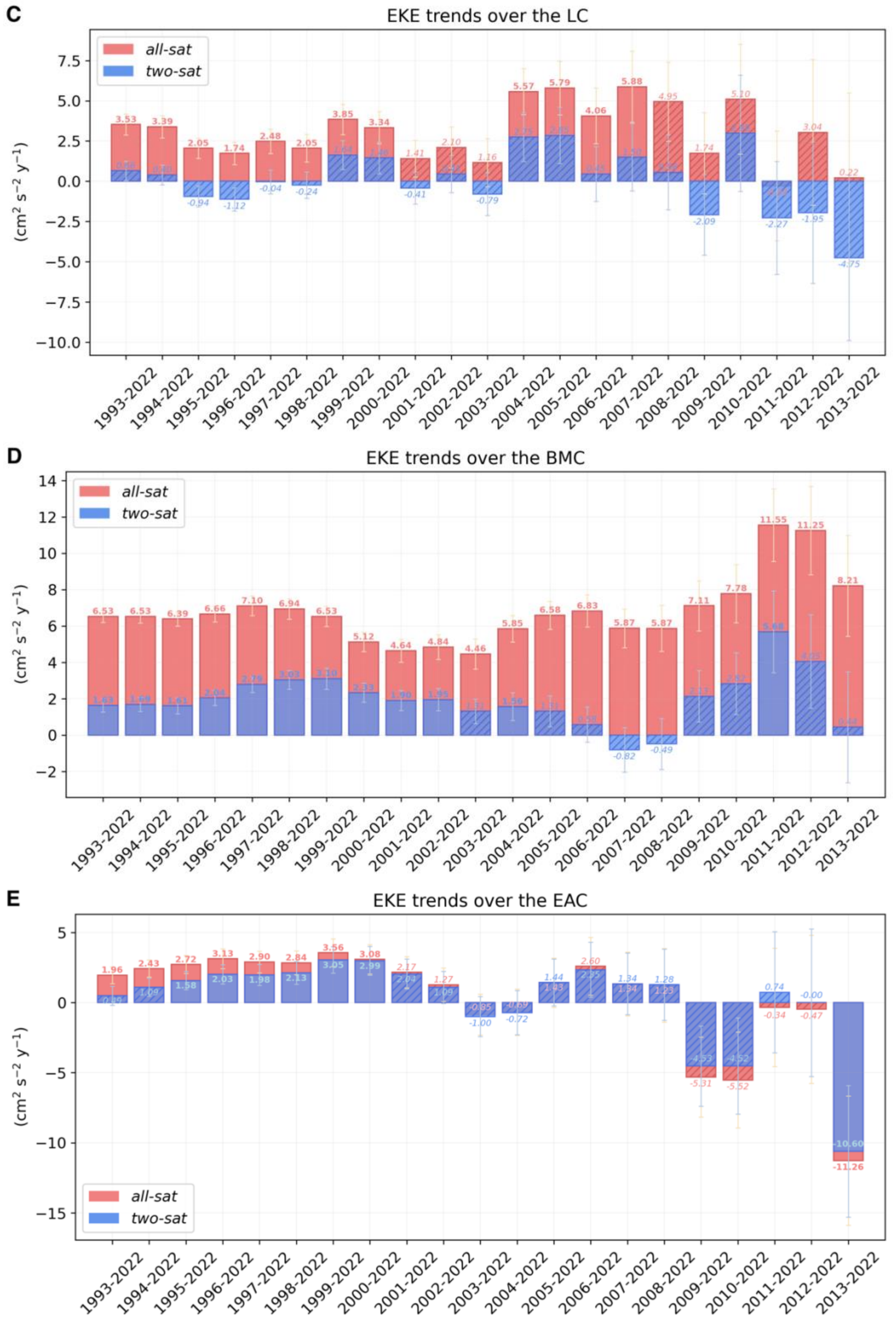

Figure S6. (continuation)

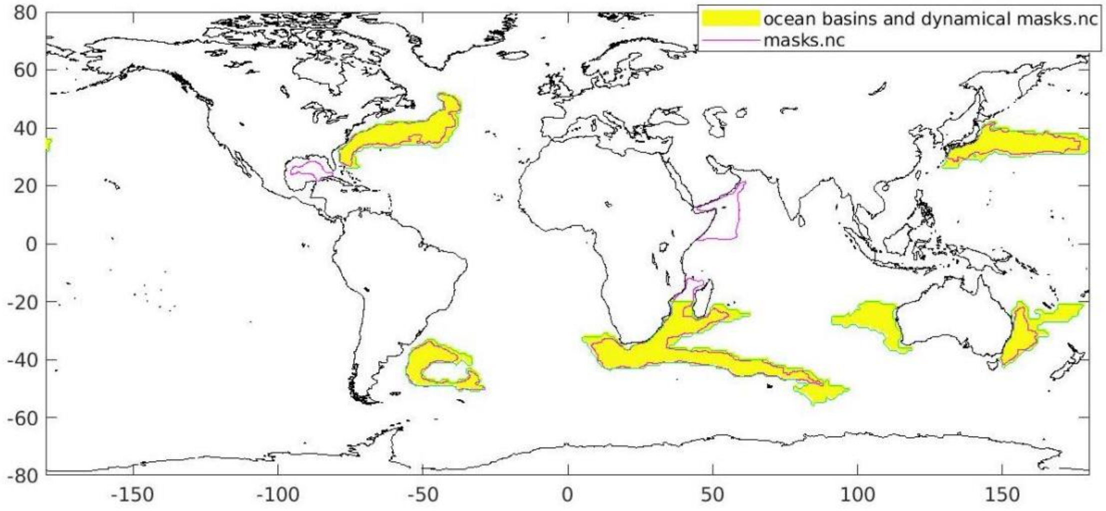

Figure S7. High EKE regions. Masks of the high EKE regions used in our study (pink contours) and used by Martínez-Moreno et al. (2021) (yellow regions).

	Area (km^2)	Mean EKE ($cm^2\ s^{-2}$)	
		all-sat	*two-sat*
Global	$3.28 \cdot 10^8$	224.83	227.09
High EKE	$1.65 \cdot 10^7$	1018.12	1003.62
Tropical	$4.39 \cdot 10^7$	330.51	354.68
Residual	$2.77 \cdot 10^8$	152.73	152.46
GWSE	$1.90 \cdot 10^6$	1179.16	1133.10
AC	$5.92 \cdot 10^6$	982.23	974.27
KE	$2.72 \cdot 10^6$	1043.04	1028.11
GS	$2.80 \cdot 10^6$	1078.97	1061.14
LC	$5.07 \cdot 10^5$	1133.63	1124.19
BMC	$1.56 \cdot 10^6$	838.23	837.05
EAC	$1.12 \cdot 10^6$	921.61	914.49

Table S1. Area and mean EKE over each region of study. This table shows the total area of each study region (Area) and the area-weighted mean of the temporally averaged EKE computed over the period 1993-2022 from the *all-sat* and *two-sat* altimetric products (Mean EKE). The regions analyzed include the global ocean (Global), the high EKE regions (High EKE), the tropical band (Tropical), the global ocean excluding the high EKE regions and the tropical band (Residual), and each specific high EKE region: Gulf Stream (GS), Kuroshio Extension (KE), Agulhas Current (AC), Brazil Malvinas Confluence region (BMC), Loop Current (LC), Great Whirl and Socotra Eddy in East Africa (GWSE), and East Australian Current (EAC).
